# Semiconductor Nanocrystals Hybridized with Functional Ligands: New Composite Materials with Tunable Properties

**DOI:** 10.3390/ma3010614

**Published:** 2010-01-22

**Authors:** Matthew McDowell, Ashley E. Wright, Nathan I. Hammer

**Affiliations:** Department of Chemistry & Biochemistry, The University of Mississippi, Coulter Hall 113, MS 38677 USA; E-Mails: doddmcdowell@gmail.com (M.M.); amevans4@olemiss.edu (A.W.)

**Keywords:** quantum dots, composite material, hybrid nanostructures, review, structural and functional properties, synthesis, ligand

## Abstract

Semiconductor nanocrystals hybridized with functional ligands represent an important new class of composite nanomaterials. The development of these new nanoscale building blocks has intensified over the past few years and offer significant advantages in a wide array of applications. Functional ligands allow for incorporation of nanocrystals into areas where their unique photophysics can be exploited. Energy and charge transfer between the ligands and the nanocrystal also result in enhanced physical properties that can be tuned by the choice of ligand architecture. Here, progress in the development and applications involving this new class of composite materials will be discussed.

## 1. Introduction

**Figure 1:**
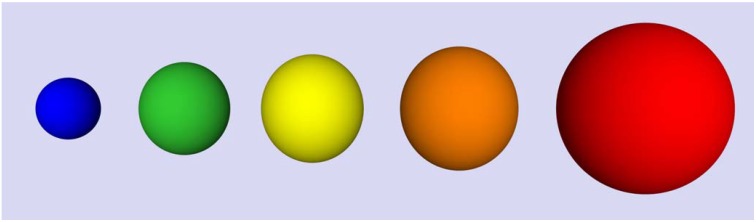
Cartoon illustrating that emission from quantum dots is dependent on the size of the particle. Larger quantum dots emit longer wavelength radiation. For CdSe, the diameter of the quantum dots in the figure would range from approximately 2.0–5.5 nm [1,2,3].

Since their initial development in the 1980s, semiconductor nanocrystals (more commonly referred to as quantum dots) have garnered a great deal of attention due to their uniquely useful properties. Their tunable optical and electronic properties make them ideal building blocks in nanoscale photonic, photovoltaic, and light-emitting diode (LED) device applications. This includes their growing use in solar cell applications, for quantum dot lasers, as sensors, single photon sources, and for quantum information processing. The robust photoluminescence of quantum dots also makes them very attractive for probing dynamics and structure in biological systems at the single molecule level, especially when compared to organic fluorophores. An important attribute of quantum dots is their rather straightforward engineering for specific applications. They are quantum systems, with properties that evolve with the size of the particle as illustrated in Figure 1. By changing their size, the optoelectronic properties can be fine tuned to match specific applications. Organic ligands that can be engineered for specific purposes are bound to the surface of quantum dots, creating this unique class of composite material. The surface bound ligands largely dictate the solution properties of quantum dots and their miscibility in various media, including organic solvents, water, and polymer films. The ability to control this surface functionality has been the subject of considerable research in recent years, and dictates the usefulness of quantum dots in different applications.

## 2. Synthesis and Properties of Quantum Dots

The variety of quantum dots that can be synthesized continues to grow. The most widely utilized quantum dots are II-VI semiconductor nanocrystals such as CdSe, CdS, CdTe, ZnSe, ZnS, and ZnTe. Other classes such as III-V (including InP, GaP, GaInP2, GaAs, and InAs) and IV-VI (including PbS, PbSe, and PbTe) have also been synthesized and their photophysical properties characterized. Nozik recently published an excellent review on the subject with many references [4]. The synthesis of many of the quantum dots listed above is rather straightforward and usually relies on the controlled nucleation and growth of the nanoparticles in solution. The choice of solvent and the ligands that are present in the reaction can have a dramatic effect on the shape and properties of the resulting nanoparticles [5,6,7,8,9,10,11,12,13,14,15]. A review by Yin and Alivisatos details these effects and the role of ligands in the synthetic process [8]. High quality and nearly monodisperse cadmium selenide quantum dots can be synthesized utilizing tri-*n*-octylphosphine oxide (TOPO) and trioctylphospine (TOP). These compounds (TOPO and TOP) have been shown to provide the most controlled growth conditions. Prior to the use of TOPO, CdSe nanocrystals were prepared by using organometallic reagents in inverse micellar solution [16]. Bawendi and co-workers showed that injection of the metal-organic precursors into a hot (~150–300 °C) reaction mixture with TOPO and TOP as the solvent solution resulted in a short burst of homogeneous nucleation and a narrow size distribution [1]. The resulting nanocrystals have TOPO ligands bound to their surface. Size-control in this synthetic procedure is achieved by varying the reaction temperature and the initial precursor concentration. Since TOPO serves as the primary surface ligand, the nanoparticles obtained by this method are soluble in hydrophobic solvents such as toluene and hexanes. The synthesis of III-V and IV-VI quantum dots also relies on nucleation and controlled growth, although the synthesis can take up to several days. Epitaxial growth is another method that can be employed for quantum dot synthesis, although the types that can be synthesized are limited.

The protection of the nanocrystal surface is an important consideration in quantum dot synthesis. Although the nanocrystal surface is usually covered with a variety of ligands (such as TOPO) from the synthetic process, defects such as dangling selenide bonds (in CdSe quantum dots, for example) serve as charge carrier trap sites, and have been associated with blinking and less than optimal device performance [17]. The objective of increased brightness of photoluminescence (quantum yield) has led to efforts of passivating surface defects with organic or inorganic ligands, either during the synthesis or afterwards. Talapin *et al.* showed that incorporation of alkylamines, particularly hexadecylamine (HDA), into the synthesis led to much improved quantum yields (as high as 50%) [9]. This result was attributed to the passivation of the cadmium selenide surface defects with the HDA ligands. It is generally very difficult, however, to simultaneously passivate both anionic and cationic surface sites by organic ligands because there would always remain some dangling bonds [18]. For this reason, capping of the nanocrystal with additional layers of semiconductor material is often an important step in quantum dot synthesis. A common example includes CdSe quantum dots capped with a shell of ZnS to form CdSe/ZnS nanoparticles [2]. These additional layers have a wider bandgap and the resulting quantum dots exhibit much higher luminescence efficiencies. In the case of zinc sulfide (CdSe/ZnS) [9,19,20] or cadmium sulfide (CdSe/CdS) [18,21,22] coated cadmium selenide quantum dots, much improved photoluminescence properties and photostability is observed with quantum yields of up to 85% in solution [22]. Sashchiuk, *et al.* synthesized both PbSe quantum dots passivated with organic ligands and PbSe/PbS core shell quantum dots. As in the case of CdSe quantum dots, the PbSe/PbS core shell quantum dots showed a substantial increase in photoluminescence compared to the organic passivated [23]. However, these inorganic surface modifications create new hybrid systems with properties which are dependent on both core and shell materials. In addition, the shell sometimes serves as an insulating medium that limits electronic communication with surface ligands or the environment, which may result in undesirable or unpredictable device performance.

**Figure 2:**
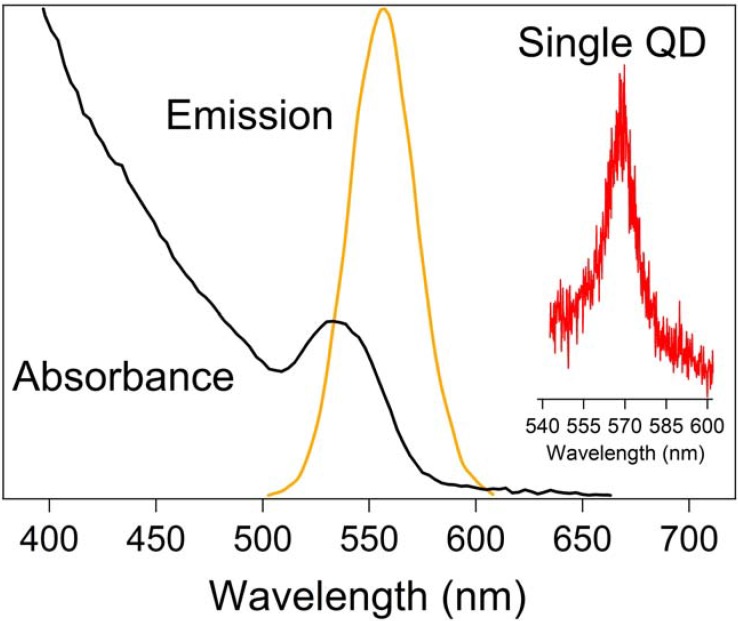
Absorbance and emission (λ_ex_ = 350 nm) curves for water soluble CdSe quantum dots. The inset shows the emission spectrum from a single quantum dot excited with the 457 nm line of an Ar^+^ laser.

The optical properties of quantum dots are unique and depend primarily upon the material employed (such as CdSe versus PbS, for example) and the size of the system. They are quantum confined systems with discrete energy levels as illustrated in Figure 1 [24]. When illuminated by light, excitons (electron-hole pairs) are formed that span the size of the nanocrystal. CdSe quantum dots are very popular for many applications because they absorb and emit light in the visible spectrum. Their emission ranges from approximately 400 nm for a diameter of 1.2 nm to close to 700 nm for >12 nm quantum dots [1]. This makes them very useful in biological applications such as visualizing and tracking molecules using photoluminescence. For example, shown in Figure 2 are the absorption and photoluminescence spectra of 3.8 nm water soluble CdSe/ZnS quantum dots obtained from Evident Technologies. We obtained these spectra with conventional UV/Vis and fluorescence spectrometers. Whereas the absorption profile extends into the ultraviolet, the emission spectrum is rather narrow and centered around 560 nm. Included in the inset is the emission spectrum of a single quantum dot, which when compared to the bulk emission spectrum, is extremely narrow. The single quantum dot was excited with the 457 nm line from an Ar^+^ laser and the emission spectrum was obtained at room temperature using an inverted microscope and Princeton Instruments Photonmax CCD camera. Other types of quantum dots exhibit the same size-dependent optical properties, but where the absorption and emission occur in the electromagnetic spectrum depends on the band gap of the material. For example, InP quantum dots emit over a range of 600 nm for 2.6 nm to 800 nm for 6 nm quantum dots [4]. PbS, PbSe, and PbTe quantum dots have emission spectra that reach into the infrared and can be tuned to match the important telecommunications region of around 1.5 μm.

In quantum dots hybridized with the appropriate organic ligands, scientists have at their disposal composite nanoscale building blocks that can emit light over narrow ranges when excited by either photons or electric current [25]. This is compared to conventional organic dyes which possess broad emission spectra and narrow absorption profiles. Quantum dots also allow for long-term observation of tagged molecules with less photobleaching than seen with conventional organic dyes thus making them the fluorophore of choice in many applications. The synthetic route taken and the choice of ligands, however, can have profound effects on the resulting photophysical properties of these new inorganic-organic hybrid materials [26,27,28]. In fact, the addition of a single ligand molecule to the quantum dot surface can have a dramatic effect on the resulting properties [29].

## 3. Ligand Exchange and Design

**Figure 3:**
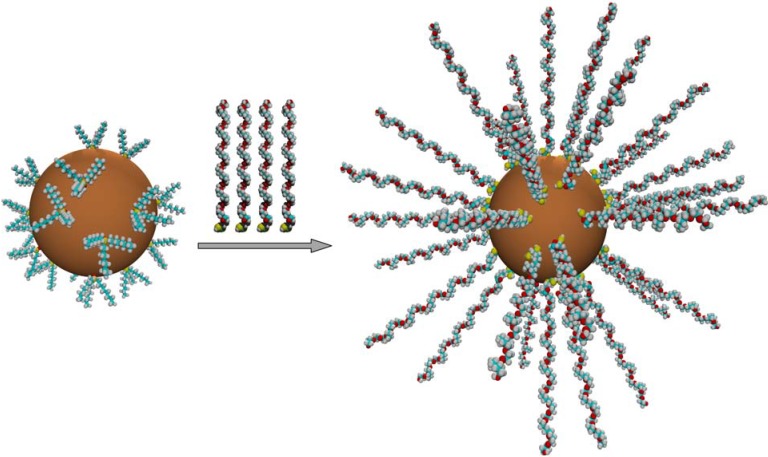
Cartoon schematic illustrating the process of ligand exchange. Organic ligands such as TOPO are exchanged for more desirable ligands such as those incorporating poly(ethylene glycol) [31].

When semiconductor nanocrystals are synthesized in solution, surface-bound ligands remain. Hydrophobic ligands such as TOPO render quantum dots soluble only in organic solvents. There are a few approaches that have been developed to address this solubility issue – which is critical for the use of quantum dots in biological and other aqueous applications. One method is ligand exchange, in which the original ligands are replaced with the ligand of choice. This process can give quantum dots solubility in hydrophilic and polar solvents such as water and can also reduce problematic aggregation of the individual quantum dots in solid state application. Ligand exchange can also make quantum dots amenable to certain secondary chemical reactions. Ligand exchange typically employs a functional group with a high affinity for binding to the surface of quantum dots (such as sulfur or phosphorous), a spacer group (such as an alkyl or aryl chain), and a functional group or chain that possesses the chemical property of interest. An example ligand exchange process is illustrated in Figure 3. Thiols have proven useful in ligand exchange due to their high affinity for the particle surface. For example, Chan and Nie used mercaptoacetic acid to make CdSe/ZnS quantum dots soluble in aqueous solution by taking advantage of the fact that the mercapto group would easily bind to a zinc atom [30].

Ligand exchange has its limitations, however, with inefficient loading of the desired ligands onto the quantum dots and poor long-term stability due to oxidation [32,33]. Alternatives to thiols include carbodithioates [34], dendrons [35,36], peptides [37], oligomeric [38] and polymer [39] phosphines, and amines [40,41,42]. In the case of carbodithioates, nearly 100% exchange has been achieved with the initial TOPO surface ligands [34]. In the case of amines, significant increases in quantum efficiency compared to TOPO have been reported using poly(allylamine) (54%) [40] and primary amines (70%) [41] due to greater passivation of surface trap sites. Wuister, *et al.* also demonstrated a significant quantum efficiency enhancement in CdTe quantum dots using ligand exchange by replacing TOP and dodecylamine ligands with either aminoethanethiol HCl or mercaptopropionic acid. In that case a quantum efficiency of 60% was reported [43]. Using computational methods to model the interactions of ligands with the nanocrystal surface can also aid in the development of more successful ligand exchange protocols using already developed ligand architectures [44,45,46]. For example, Puzder, *et al.* studied the interaction of phosphine oxide, phosphonic acids, carboxylic acids, and amine ligands with CdSe quantum dots and showed that the dominant interactions present were between oxygen atoms in the ligands and cadmium atoms on the nanoparticle surfaces. [44] Pong, *et al.* studied the binding of alkyl thiols to ZnS theoretically and used their results to dramatically improve the ligand exchange process [45].

Employing multidentate ligands helps create more stable interactions with the nanoparticles. For example, Uyeda *et al.* employed ligand exchange using poly(ethylene glycol) ligands of various lengths with two thiol linkages [31]. Other examples of polydentate ligand architectures include using dimethylaminoethyl methacrylate [47,48], thioalkyl acid ligands [49], and modifying polymer ligands to incorporate multiple thiol and amine groups along the ligand backbone [50]. In the last case, the goal was to reduce the hydrodynamic size of the nanoparticle without losing their advantageous photophysical properties so that they could be employed for specific applications. Despite the possible drawbacks of ligand exchange, such as the possibility of loss of fluorescence and an incomplete ligand coverage, this rather straightforward and facile method is necessary in most cases, as the high-temperature of nanocrystal reaction mixture is not compatible with most organic functional groups. Quantum dots can also be encapsulated by a shell of material such as a polymer, micelle, or bead that makes them more soluble in particular media [51,52]. Such encapsulation increases the volume of the quantum dot-based material, which may not be desirable in some applications, such as biosensors and live cell imaging.

In 2003, Alivisatos and Fréchet developed a pentathiophene surfactant-based ligand with the goal of facilitating charge transfer between the nanocrystal and an organic semiconducting matrix [53]. The next year, Advincula used ligand exchange to create composite systems composed of a quantum dot core and oligothiophene dendron ligands. In 2005, Pron compared the properties of TOPO covered quantum dots with those covered with oligoaniline ligands [54]. In that case, they discovered that the ligands significantly affected the spectroelectrochemical properties of the quantum dots. Thus, by employing chemically active ligands one could not only change the solubility of the quantum dots but also affect their chemical and photophysical properties. More recent efforts have concentrated on replacing TOPO as the solvent during the synthesis with a ligand that possesses functionality that allows for subsequent surface grafting without the need for ligand exchange. For example, Wang *et al.* demonstrated such a system that allowed for the easy addition of antibodies to the quantum dot surface without the need for ligand exchange for use in cell labeling. [55]

**Figure 4:**
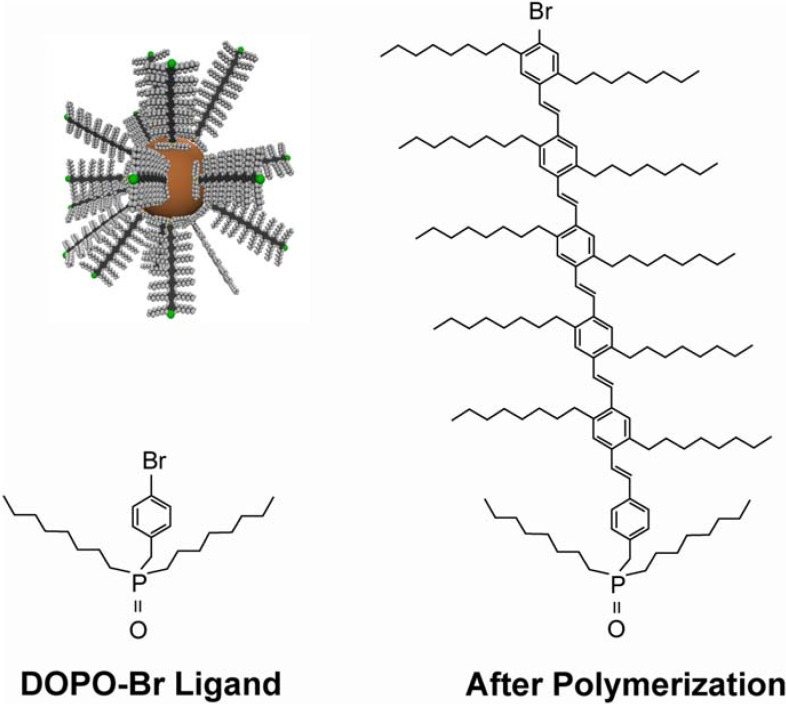
Top Left: CdSe quantum dot with oligo-(phenylene vinylene) ligands. Bottom Left: DOPO-BR ligand prior to polymerization reaction. Right: oligo-(phenylene vinylene) ligand after polymerization from DOPO-Br.

In 2004 Emrick and co-workers started to employ phenyl bromide-functionalized dioctylphosphine oxide (DOPO-Br) during the quantum dot synthesis in place of TOPO [56]. DOPO-Br proved to be stable in the high temperature reaction conditions of quantum dot growth, giving DOPO-Br covered CdSe nanocrystals. They then used Heck-type coupling to synthesize poly- or oligo-(phenylene vinylene) (PPV or OPV) ligands *directly* from the functional quantum dots. Shown in Figure 4 is a DOPO-Br ligand prior to polymerization, the resulting OPV ligand, and a cartoon schematic of composite CdSe-OPV nanostructure. The photophysical properties of CdSe-OPV nanostructures have recently been studied in much detail and will be highlighted in more detail as an example composite system below.

The length of the OPV ligands on the CdSe surface in CdSe-OPV nanostructures can be tuned so that the fluorescence emission spectrum overlaps the absorption spectrum of the quantum dot cores. Whereas the fluorescence emission spectra of bulk mixtures or blends of OPV and CdSe quantum dots only reveal emission from both components, in the case of the CdSe-OPV nanostructures, however, virtually no OPV emission is observed [57]. This result implies quenching of the OPV emission when the ligands are directly connected and coupled to the quantum dot and that enhanced energy transfer is taking place. In an effort to determine the effects of the bound conjugated organic ligands on individual quantum dot photophysics, single molecule spectroscopic measurements were performed on the CdSe-OPV nanostructures. Significant differences were apparent when comparing the photoluminescence emission spectra of the CdSe-OPV nanostructures and the DOPO-Br covered or TOPO covered ZnS capped CdSe quantum dots. Whereas the emission intensity from both the DOPO-Br covered and ZnS capped quantum dots was intermittent, the emission from the single CdSe-OPV nanostructure was continuous. This observation led Emrick and Barnes to investigate the possibility of charge transfer between the CdSe quantum dots and the bound ligands.

Charge transfer in the form of electrons from phenylene vinylene to CdSe quantum dots has been shown to be energetically favorable because of the relative electron affinities of the two species [58]. OPV ligands would therefore be expected to serve as good electron donating ligands for quantum dots and in principle suppress quantum dot blinking. The OPV ligand coverage was correlated to the fluorescence properties for a large number of nanostructures using atomic force microscopy (AFM) and these results were compared to conventional ZnS-capped CdSe quantum dots [59,60]. Whereas the average emitting time was observed to be near 40% for the ZnS-capped quantum dots, most of the CdSe-OPV nanostructures emitted light nearly 100% of the time. Most interesting is the fact that the larger the height signature (more OPV ligands), the longer the period of light emission and the greater the blinking suppression. This implies that the ligands were affecting the CdSe quantum dot in an additive manner—the more ligands, the greater the enhanced properties. More recently, Emrick and Barnes have characterized the quenching of the fluorescent lifetimes of the quantum dot cores by the polymer ligands [61,62] as well as described the composite nanostructures in terms of linear dipoles through polarization anisotropy measurements [63].

## 4. Quantum Dots in Biological Imaging, Tracking, and FRET

The most prolific use of quantum dots has come in the form of biological applications, which includes both imaging of tissue and tracking of biomolecules [30,31,32,36,37,39,51,64,65,66,67,68,69,70,71,72,73,74,75,76,77,78,79,80,81,82,83,84,85,86,87,88,89,90,91,92,93,94,95,96,97,98,99,100,101,102,103,104,105]. As pointed out earlier, when compared to conventional organic dyes, quantum dots are much more stable and have much more narrow emission profiles. The key factor in the use of quantum dots in biological media is getting them to be water soluble. CdSe quantum dots created with TOPO ligands, for example, are insoluble in water. Therefore, quantum dots must be either synthesized with water soluble ligands or ligand exchange must be employed. As discussed earlier, water soluble thiols are an attractive choice using ligand exchange because of their high affinity for the nanocrystal surface [18,33,43]. For example, Wuister, *et al.* transferred CdTe quantum dots into water by the use of aminoethanethiol HCl and mercaptopropionic acid ligands [43]. There are many alternatives to thiols, however such as water soluble phosphine oxide polymers [39]. Colvin compiled a detailed review of the various approaches at creating water soluble quantum dots for biomolecular applications and includes a number of references on the subject [68].

**Figure 5:**
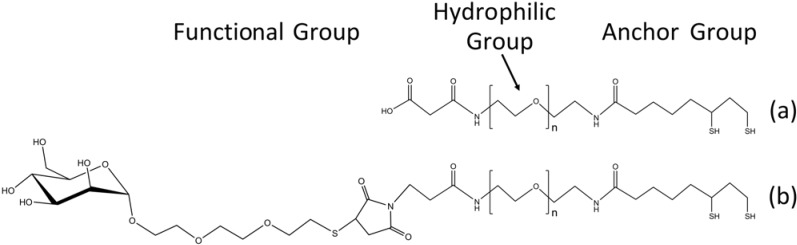
Water soluble functional ligands for biological applications consisting of a bidentate linkage group that connects to the quantum dot surface, a poly(ethylene glycol) chain for solubility, and a functional end group consisting of (a) a carboxylate group [96,99] or (b) D-mannose [105].

Recent efforts have concentrated on engineering ligands that incorporate a high degree of functionality for biological applications. Some examples of these new functional ligands are shown in Figure 5. Murcia, *et al.* recently introduced functional ligands based on 2-(2-aminoethoxy)ethanol for the attachment of lipids [94]. Susumu, *et al.* introduced functional ligands composed of a dihydrolipoic acid linker that attaches to the quantum dot surface, a poly(ethylene glycol) chain that makes the ligands water soluble, and a functional end group that can be used to derivative the quantum dot for the purpose at hand [99]. Liu, *et al.* also presented a family of water-soluble quantum dots incorporating heterobifunctional ligands [96]. These ligands were also composed of dihydrolipoic acid, a short poly(ethylene glycol) spacer, and an amine or carboxylate end group. In their contribution they covalently attached streptavidin to their ligands and imaged epidermal growth factor receptors in live cells. Others have also recently employed the use of poly(ethylene glycol) based ligands for biocompatibility. Yildiz, *et al.* showed that quantum dots hybridized with such ligands were not cytotoxic and in fact the localization of the introduced quantum dots could be controlled by the length of the ligands [103]. Kikkeri, *et al.* recently employed such ligands capped with D-mannose, D-galactose, and D-galactosamine for the study of carbohydrate-protein interactions [105].

**Figure 6:**
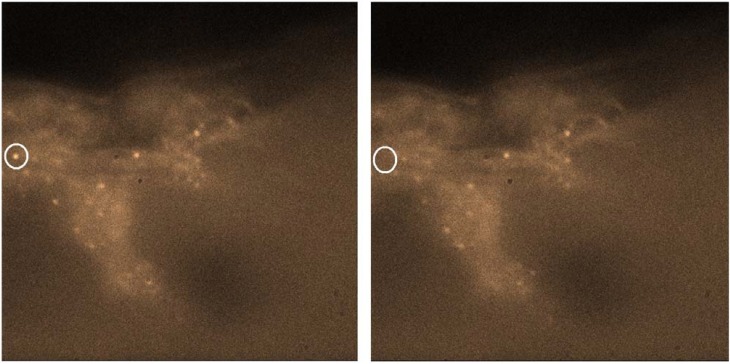
Transverse cuts of porcine skin cells (Top = top of skin) imaged with water soluble quantum dots imaged at different times. Here, single quantum dots can be observed in the cellular structure with one (circled) blinking.

An example of the use of quantum dots for biological imaging is shown in Figure 6 are porcine skin cells imaged using the same water soluble quantum dots described in Figure 2. For these images the 457 nm line of an Ar^+^ laser was used to excite the quantum dots in the skin. The goal of this study was to determine where the quantum dots migrated to in the skin and how deep they can travel. The effect of the surface ligands on this mobility is also expected to play a big role and has important consequences in the use of quantum dots in drug delivery. The images were collected with a Nikon TE2000U inverted light microscope and detected with a Princeton Instruments Photonmax CCD camera. In this study, the emission spectrum was obtained at the same time as the image – thus confirming that the emission did indeed originate from the quantum dots [106].

The design of the quantum dot ligands as linkages to the biomolecules to be tracked or imaged is an important key step in the development of a quantum dots as successful probes. For example, Bawendi has used dihydrolipoic acid as an electrostatic linker to image proteins [67]. The choice of ligand is key to the biomolecular application at hand and Weiss has recently reviewed biologically relevant applications of a great number of quantum dot—ligand combinations [66]. One molecule that has seen much success is streptavidin. For example, streptavidin coverage has proven very attractive for its use in combination with biotinylated proteins and antibodies. Imaging biomolecules at the single molecule level is also a very active area of research today. Whereas conventional ensemble measurements detail the average properties of the system under study, single molecule measurements can reveal the hidden heterogeneity and the time dependent properties of single molecules. Mattoussi has recently written a nice review with many references on this very active area of research [69].

There has also been a great deal of recent interest in energy transfer involving quantum dots and fluorescently-labeled biomolecules [102,107,108,109,110,111,112,113,114,115,116,117,118,119,120]. Fluorescence resonance energy transfer (FRET) from an excited state donor to a ground state acceptor occurs when the donor’s emission spectrum overlaps the acceptor’s excitation spectrum. The spatial distance between the two components determines the efficiency of the energy transfer process [121]. Mattoussi recently reviewed many of the studies of FRET involving quantum dots and biological systems [120]. Bawendi and co-workers first demonstrated FRET energy transfer involving mixtures of different sizes of quantum dots [122]. Emission from smaller, higher energy, quantum dots decreased as emission from the larger, smaller energy level spacing, quantum dots increased. The first observation of FRET between quantum dots and biological molecules was made in 2001 by Van Orden and co-workers [107]. In that study, quantum dots with biotinylated bovine serum albumin ligands interacted with streptavidin that was labeled with a rhodamine dye. Emission from the dye was observed to increase as emission from the quantum dot diminished. Kotov and co-workers reported energy transfer to quantum dots from native tryptophan molecules in an interacting protein [108]. Other interesting FRET applications include interactions with metalloproteins [116] and DNA [114]. Mattoussi reported spectrally resolved energy transfer between quantum dots and fluorescently tagged proteins [118]. Both Liu, *et al.* [96] and Susumu, *et al.* [99] demonstrated the versatility of their functionalized ligands using FRET between the quantum dots and their ligands and Ha recently demonstrated FRET between single quantum dots and their ligands [111].

## 5. LEDS and Lasers

**Figure 7:**
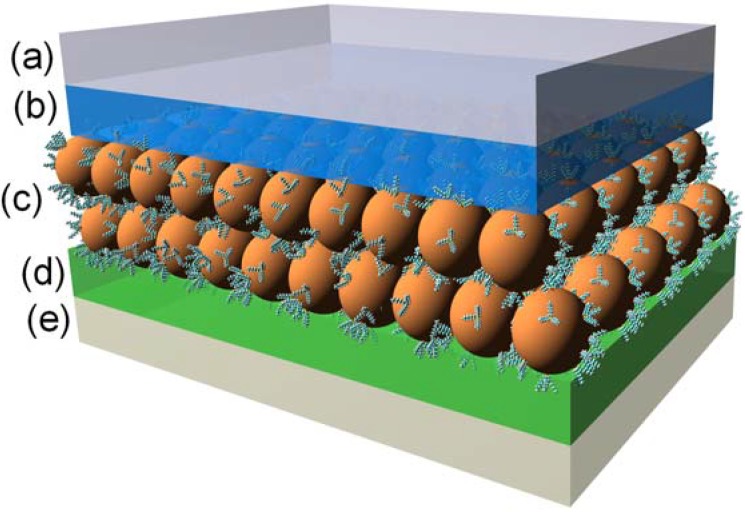
Schematic of a light emitting device incorporating quantum dots: (a) cathode, (b) electron transporting layer, (c) organic ligand functionalized quantum dots, (d) hole transporting layer, (e) anode.

The optoelectronic properties (broad absorption spectrum and narrow emission profile) of quantum dots make them very attractive building blocks for a number of solid state applications. The creation of light emitting diodes (LED’s) from semiconductor nanocrystals is a natural development due to the quantum nature of the material. The emission color can be finely tuned by changing the size of the quantum dot and the quantum dot material. Alivisatos introduced a hybrid inorganic/organic LED in 1994 in which holes from a PPV layer joined with electrons from CdSe nanocrystals to create light emission [123]. The next year, Bawendi introduced a similar device composed of CdSe quantum dots and polyvinylcarbazole (PVK) that was tunable (by changing the quantum dot size) over the range of 530–650 nm [124]. Over the last fifteen years a number of devices have been reported that operate on similar principles [125,126,127,128,129,130,131,132,133,134,135,136,137]. One of the difficulties involved in constructing devices from quantum dots is creating electrical contacts to electrodes that allow charge transport while at the same time preventing quantum dot aggregation. The early photovoltaic devices suffered from aggregation of the particles, which is far from optimal for facilitating charge separation and transport to the respective electrodes. More recent efforts have been directed at directly linking conjugated organic polymers to the surface of the quantum dots such as in composite CdSe-OPV nanostructures described above. A cartoon schematic of a device incorporating such functionalized quantum dots is shown in Figure 7. This direct connection has been shown to eliminate aggregation effects [56] and is expected to lead to much higher device efficiencies. An excellent example of such a device was recently introduced by Zorn *et al.* [138]. In that case, the authors attached block copolymer ligands to CdSe/ZnS quantum dots. The copolymers consisted of both semiconducting blocks and reactive functional blocks that contain multidentate thiol-based anchor groups. The key to the success of this architecture is that by being bound to the quantum dot surface, the semiconductor ligands improved hole injection into the quantum dots. This, in turn, resulted in improved device performance (increased electroluminescence intensity and quantum efficiency) compared with a similar device containing unmodified quantum dots.

The development of lasers composed of quantum dot lasers is expected to lead to devices that are at the same time inexpensive and tunable. By changing the size of the quantum dot building blocks, the color of the laser could be easily selected. In addition, lasers developed using quantum dots are expected to not be affected by temperature, have lower lasing thresholds, and exhibit a gain profile concentrated into a much narrower spectral region than the bulk material [139]. Bawendi demonstrated optical gain and stimulated emission in 2000 using CdSe quantum dots, developed a distributed feedback laser in 2002 [140], and has made subsequent improvements since that time [141,142,143]. In addition to these, there have been a number of other quantum dot based lasers developed over the last decade [144,145,146,147,148,149,150,151,152,153]. In 2005, Bawendi demonstrated that the passivation due to surface ligands played a key role in lasing efficiency at blue wavelengths. This resulted in stable room-temperature lasing over long periods of continuous excitation. Choosing the best surface ligands for quantum dot laser building blocks also allows for greater miscibility in the laser host medium and should prove to be important in future quantum dot laser developments.

## 6. Solar Cells

Perhaps the greatest promise that functionalized quantum dots offer is that of converting light energy to electrical energy in photovoltaic or solar cells. The vast majority of success in the development of solar cells has come when using TiO_2_-based devices. However, ultraviolet light is required to excite charge carriers in TiO_2_, making it inconvenient for use in solar cells. Sensitization using organic dyes has proven successful in shifting the absorbance maxima of many solar cells into the visible [154,155,156]. The tunability of quantum dots allows for the same result to be achieved with the added benefits of increased photostability and extremely high quantum yields. There have been a number of solar cells developed using quantum dots as sensitizers [157,158,159,160,161,162,163,164,165,166,167,168,169,170,171,172,173,174,175,176]. Key issues in the use of quantum dots in solar cell applications vital to creating viable devices are charge separation and improving the delivery of electrons to TiO_2_. A solution to these problems involves developing a charge conduit between the quantum dots and the large band gap semiconductor. Both fullerenes [177] and nanotubes [178] have been suggested as such charge conduits. As in the case of other device applications, the miscibility of the quantum dots is dictated by their surface ligands and the same is true when quantum dots are coupled to such charge conduits. However, if the ligands on the quantum dots themselves could serve as both the dispersive agent and the charge conduits then one would have a fully integrated building block without the need for hybrid blends. Such as system was recently investigated by Majima [166] and is the basis for the development of the CdSe-OPV nanostructures developed by Emrick [56,57,59,60,61,62,63]. Sargent also recently reported that the use of thiol ligands on PbS quantum dots resulted in much greater photovoltaic device efficiency in the infrared region of the electromagnetic spectrum [175,179]. Research into such ligand-based composite systems is likely to increase over the next few years as the desire for inexpensive quantum dot solar cells intensifies.

## 7. Summary

Through careful engineering, composite nanostructures composed of quantum dot cores surrounded by functional organic ligands are being developed that offer a number of unique advantages. Tunable photophysics, selective solubility, enhanced energy transfer, charge transfer, and effective sensitization have all been realized in recent years with applications in areas ranging from single biomolecule tracking to solid state device fabrication. In the coming years, progress in this area is likely to continue with a number of significant applications realized.
